# Study of the mechanism of change in flavonoid composition in the processing of *Chrysanthemum morifolium* (Ramat.) Tzvel. ‘*Boju*’

**DOI:** 10.1186/s13065-019-0645-0

**Published:** 2019-11-09

**Authors:** Wei Zhang, Yafeng Zuo, Fengqing Xu, Tongsheng Wang, Jinsong Liu, Deling Wu

**Affiliations:** 1 School of Pharmacy, Anhui University of Chinese Medicine, No. 1 Qianjiang Road, Hefei, 230000 China; 2 School of Chinese Materia Medica, Bozhou University, No. 2266 Tangwang Road, Bozhou, 236800 China

**Keywords:** *β*-Glucosidase, Change in flavonoid composition, *Chrysanthemum morifolium* (Ramat.) Tzvel. ‘*Boju*’

## Abstract

A form of *β*-glucosidase was isolated and purified from fresh *Chrysanthemum morifolium* (Ramat.) Tzvel. ‘*Boju*’ (*Boju*) and its enzymatic properties explored in this study. The purified enzyme and *Boju* flavonoids were reacted in a water bath to ascertain the composition of the reactants. Flavonoid glycoside and aglycon concentrations in *Boju* varied significantly depending on processing method. The concentration of flavonoid glycosides in *Boju* decreased and flavonoid aglycons increased due to heat-activation of *β*-glucosidase which hydrolyzed the flavonoid glycosides in *Boju* to aglycons.

## Background

According to differences in origin and processing methods, *Chrysanthemum morifolium* Ramat. can be categorized into ‘*Boju*’, ‘*Chuju*’, ‘*Gongju*’, ‘*Hangju*’ or ‘*Huaiju*’, as included in the 2015 edition of the Chinese Pharmacopoeia [1, 2]. “Drug Production Identification”, “Chinese Medicine Dictionary”, “Chinese Medicine” and other books state that *Boju* represents the best quality in medicinal chrysanthemum. The traditional way of drying *Boju* is to cut the whole plant and hang it to dry in the shade prior to removing the inflorescence. During our research, it was found that in order to rapidly dry a large number of medicinal materials, traditional drying methods are no longer used to dry the plants. There are two principal processing methods that are commonly used: one is to dry the *Boju* after steaming, the other is to directly dry after picking the flowers. These two methods essentially fulfil the requirements of large-scale production. However, the method of processing defines the respective medicinal properties of *Boju* [3, 4] and due to changes in the traditional processing methods of *Boju* production (such as drying, evaporation, sulfur fumigation, et al.) some of the intrinsic quality of *Boju* herbs has been lost [5–10]. Previous studies have demonstrated that different processing methods of *Boju* can affect the content of flavonoids in *Boju*. It has also been found that *Boju* extract can catalyze the hydrolysis of glycosides when ρ-nitrophenyl-*β*-d-glucopyranoside (ρNPG) is used as a substrate [7, 11]. Thus, the reasons for the changes in flavonoids in *Boju* require investigation.

Many researchers have investigated over recent years the transformation of flavonoid glycosides to aglycones by enzymatic hydrolysis and microbial fermentation. The results of Caldwell et al. [12] have shown that the isoflavone content is reduced when storage temperature is raised from 18 to 28 °C. Mao et al. [13] found that fermented soybean, in the processing of traditional Chinese medicine, changed in soybean and wood glycosides concentration due to the role of enzymes during processing, genistein being converted to daidzein in significant concentrations. Feng et al. [14] used microbial enzymes to ferment safflower, and through hydrolysis of antioxidant glycosides improved its antioxidant efficacy. Zhang et al. [15] found that hydrolysis of *Apocynum venetum* flavonoid glycosides improved the extraction of flavonoids from its leaves and thus its antidepressant activity. It has been demonstrated that the *β*-glucosidase activity of *Boju* may perform an important role in the transformation of flavones.

Considerable research has been conducted on Boju, but those studies do not explain the mechanisms of changes in its chemical composition [7, 10]. Consequently, we studied the relationship between change in chemical composition and enzymes in *Boju* in this study and found significant *β*-glucosidase activity. The aim was to purify and characterize the newly discovered *β*-glucosidase. We have also reported on the transformation of *Boju* flavones by this *β*-glucosidase. The results indicate that the flavonoid content of *Boju* depends on changes in enzymatic activity.

## Methods

### Materials

*Boju* Plants were collected from the Bozhou medicinal material market in November 2014 and identified as belonging to the genus *Chrysanthemum morifolium* (Ramat.) Tzvel. *‘Boju’* by Prof. Shou-jin Liu. Fresh *Boju* was snap frozen in liquid nitrogen and stored at − 80 °C until analyzed.

### Reagents and equipment

DEAE-cellulose-52 (Shanghai Yuanye Biotechnology Co., Ltd, China); SephadexTM G-100 (GE Healthcare, Swedish); Prestained Protein Ladder (Thermo Fisher Scientific (China) Co., Ltd., China); DW-S6L388A vertical ultra-low temperature storage box (Qingdao Haier Special Electric Co., Ltd, China); Heraeus Multifuge X1R cryogenic centrifuge (Thermo Fisher, American); 10kD ultrafiltration centrifuge tube (Millipore, American).

### Determination of the main components in different processed products of *Boju*

#### Preparation of different processed products of *Boju*

Sample 1: Fresh *Boju* flower heads were removed and steamed for 2 min then placed in an oven at 50 °C for drying. Samples 2 and 3: Fresh *Boju* flower heads were dried in a microwave oven at 700 W for 1 min or 2 min, respectively, then at 350 W until completely dry. Sample 4: *Boju* plants were hung upside down in a cool, ventilated location until completely dry prior to the flower heads being removed. Sample 5: Fresh *Boju* flower heads were placed in a cool, ventilated location until completely dry. Samples 6–11: Fresh *Boju* flower heads were placed in a constant temperature drying oven at 40 °C, 50 °C, 60 °C, 70 °C, 80 °C or 90 °C, respectively, then dried completely.

### Analysis of content

The principal components of *Boju* samples 1-11 were measured by high performance liquid chromatography (HPLC). A linear solvent gradient comprising acetonitrile (solvent A) and 0.1% phosphoric acid solution (solvent B) was used. Following the injection of 20 μL of sample, solvent A was increased from 12 to 17% over 8 min, 17% to 19% over 17 min, maintained at 19% for 5 min, then increased to 60% over the subsequent 30 min and finally to 12% within 5 min. Solvent flow rate was 1 mL/min. The HPLC system comprised a Waters 2707 autosampler and Waters 1525 Binary HPLC pumps equipped with a Waters 2498 UV/Visible detector and a reverse-phase analytical column (Agilent HC-C_18_(2)M, 4.6 mm × 250 mm, 5 μm, CA, USA) for separation. The eluted components were detected at 330 nm.

### Preparation of *Boju* for measurement of *β*-glucosidase activity

Fresh *Boju* (100 g) was soaked in 500 mL of 100 mM cold citric acid buffer at pH 4.0, a small quantity of quartz sand and 25 mL glycerol prior to being homogenized for 2 min in a blender. The slurry was slowly stirred at 4 °C for 1 h then poured into a cotton filter bag prior to vacuum filtration for removal of insoluble residue. The filtrate was centrifugated at 12,000 r/min for 20 min and the supernatant analyzed for *β*-glucosidase activity (ρNPG assay) and protein concentration (Bradford assay).

### Purification of *β*-glucosidase from *Boju*

#### Ammonium sulfate fractionation

A 500 mL volume of supernatant was concentrated to a final volume of 100 mL using dialysis. Fine ammonium sulfate was slowly dripped into the solution until the ammonium sulfate concentration was 20% (w/v) then the solution was stirred at 4 °C for 30 min. The precipitated protein was collected by centrifugation (12,000 r/min, 15 min). Likewise, the remaining supernatant was sequentially fractionated by precipitation with 30%, 40%, 50%, 60%, 70%, 80%, 90% and 100% ammonium sulfate solution. *β*-Glucosidase activity and protein content was quantified in the precipitate and supernatants obtained from those ammonium sulfate concentrations.

#### Desalination and concentration

The fraction with the highest *β*-glucosidase activity (i.e. the precipitate obtained from an ammonium sulfate concentration within the 30–80% fractions) was dissolved in 10 mL of 50 mM phosphate buffer, pH 6.0. Salt was removed from this fraction and the solution concentrated to a certain volume using an ultrafiltration centrifuge tube.

#### Purification by column chromatography

The active enzyme solution was further purified using a DEAE celluose-52 anion-exchange chromatography column. The column (3.0 cm × 25 cm) was packed with 100 mL preswollen cellulose and equilibrated with 50 mM, pH 6.0, phosphate buffer. Active enzyme solution was then loaded onto the top of the column and then eluted with the same phosphate buffer until the UV absorption value at 280 nm no longer varied. The fraction adsorbed onto the DEAE celluose-52 was eluted with a concentration gradient of NaCl from 0.2 to 1.6 M in 50 mM, phosphate buffer, pH 6.0 at a rate of 0.8 mL/min. The elution volume of each phase was 200 mL. Fractions with significant *β*-glucosidase activity were combined and concentrated to 1 mL using an ultrafiltration centrifuge tube. A gel filtration column (Sephadex™ G-100) was used for further purification. The active enzyme solution was loaded onto the column and eluted with 50 mM phosphate buffer, pH 6.0 at a flow rate of 0.5 mL/min. The eluate was collected in 4 mL fractions.

### *β*-Glucosidase measurement

*β*-Glucosidase activity was assayed with ρNPG as the substrate. A mixture of 200 μL, 15 mM ρNPG (dissolved in 0.5 M phosphate buffer, pH 6.0), and 200 μL of enzyme solution was incubated in a 50 °C water bath for 45 min, then 2.5 mL of 1.0 M sodium carbonate solution was added, and the absorbance at 400 nm measured. A standard curve of 1 to 60 μM ρ-nitrophenol solution in 1.0 M sodium carbonate was prepared in the same way. A unit of *β*-glucosidase activity is defined as the quantity of enzyme releasing 1 μmol of ρ-nitrophenol per min from the substrate.

### Enzyme purity analysis and sodium dodecyl sulfate-polyacrylamide gel electrophoresis (SDS-PAGE)

#### Enzyme purity determination

The purity of the enzyme solution obtained was determined by HPLC. Chromatographic conditions: Asahipak GF-510HQ (7.5 mmol/L × 300 mmol/L, 5 μm) column; column temperature: 25 °C; mobile phase: methanol:water ratio = 30:70; flow rate: 0.6 mL/min; injection volume: 20 μL; detection wavelength: 280 nm.

*SDS*-*PAGE* SDS-PAGE was performed as described by Ma [16]. Analysis was conducted within nonreducing conditions. A mixture of markers obtained from Thermo Fisher Scientific was used as protein molecular weight standards. Gels were visualized using silver staining, as described previously [17].

### Hydrolysis of substrates by *β*-glucosidase

To study the effect of the purified enzyme on total flavonoids in *Boju*, the flavonoids were prepared and used as a flavonoid-substrate for the enzyme assay. A 25.45 mg quantity of total flavonoid was dissolved in 10 mL of 0.1 M phosphate buffer, pH 5.0 and equilibrated in a 50 °C water bath. The catalytic reaction of the purified *β*-glucosidase was initiated by adding 200 μL of purified enzyme solution and mixing thoroughly. After incubation at 50 °C for 1 h, 1 mL of methanol was added to stop the reaction. In addition, 1 mL of methanol was added to a second purified enzyme solution to inactivate it which was used as the blank control. The reaction mixture was centrifuged to remove insoluble substances, and composition analysis of total flavonoids in *Boju* ascertained from the supernatant. Chromatography was conducted using the conditions described in “Content determination”.

## Results and discussion

### Determination of main components in different processed products of *Boju*

**Fig. 1:**
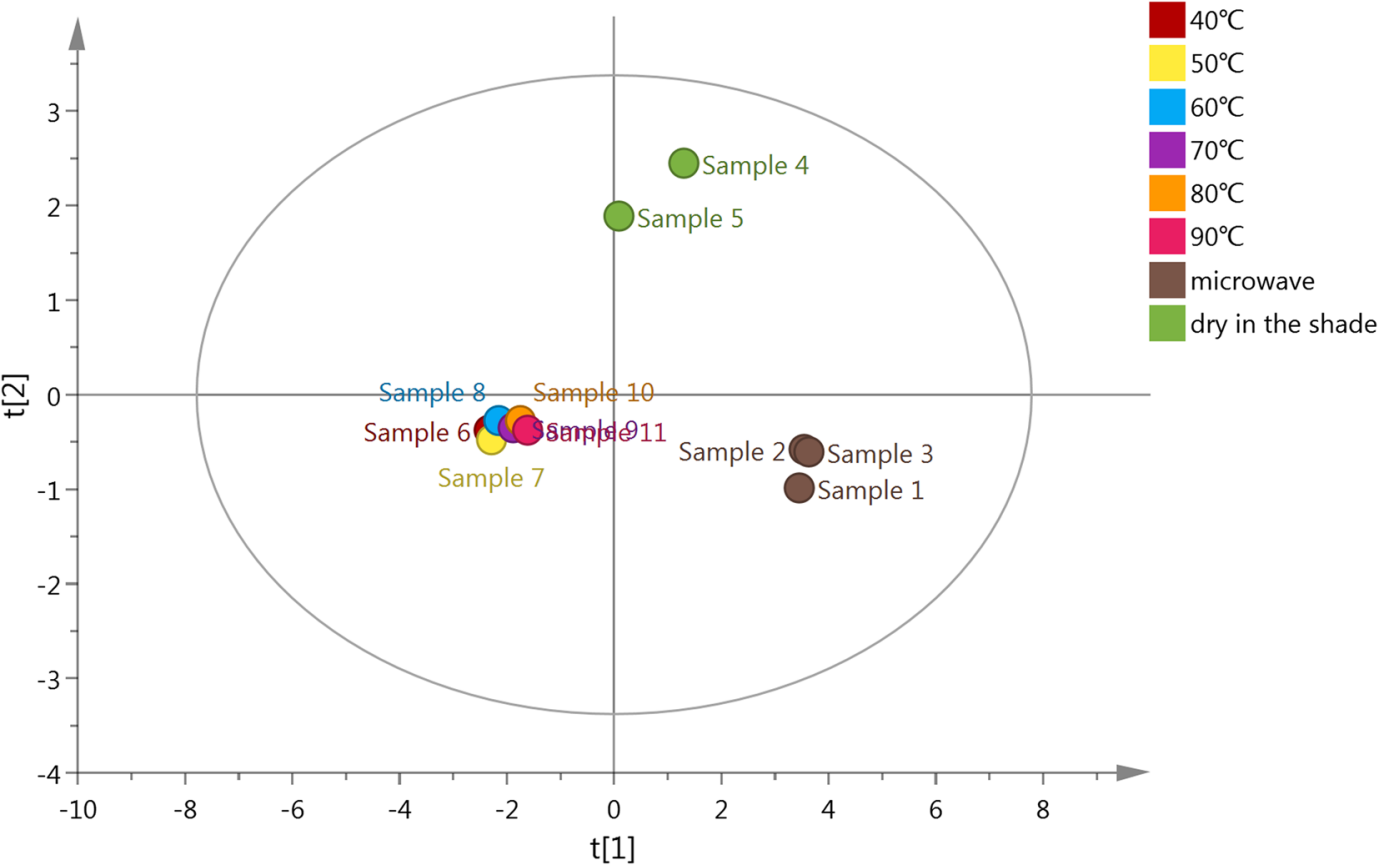
PCA results of *Boju* samples purified using different processes

**Fig. 2:**
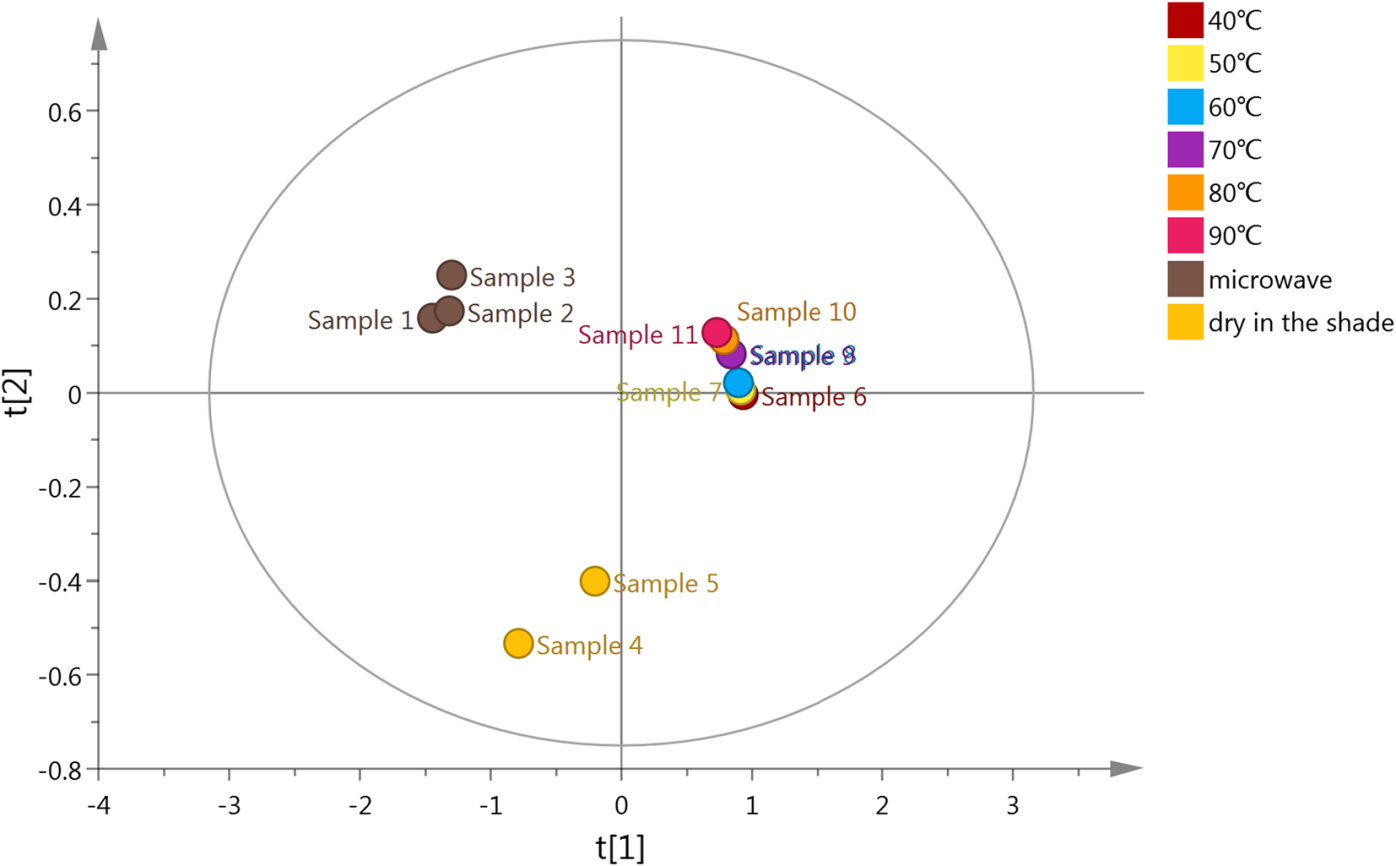
The PLS-DA results of *Boju* samples purified using different processes

**Fig. 3:**
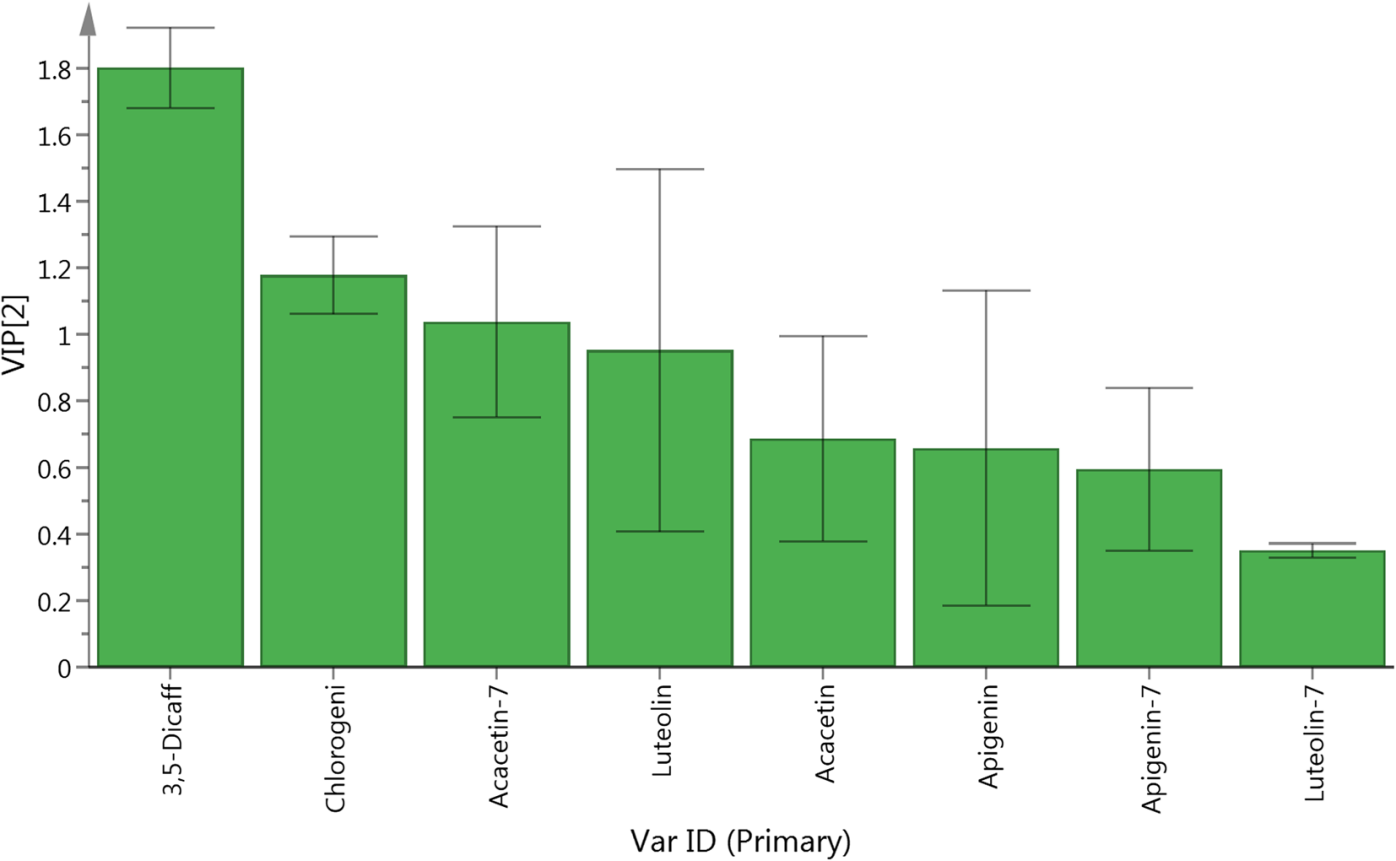
VIP Plot of 8 compositions

Principal components analysis (PCA) and partial least squares regression with discriminant analysis (PLS-DA) were used to analyze the main components within *Boju* (Table 1). PCA indicated that the different processing methods of *Boju* could be divided into three categories: group 1 (samples 1, 2, 3), group 2 (samples 4, 5) and group 3 (samples 6, 7, 8, 9, 10, 11). PLS-DA demonstrated that the components with the greatest influence on the classification of *Boju* during the drying process were 3,5-dicaffeoylquinic acid, chlorogenic acid and acacetin-7-O-glucoside, while luteolin-7-O-glucoside contributed the least (Figs. 1, 2, 3).

**Table 1:** Content of 8 components in *Boju* following different processes (%) (n = 3)

Sample	Chlorogenic acid	3,5-Dicaffeoylquinic acid	Luteolin-7-O-glucoside	Apigenin-7-O-glucoside	Acacetin-7-O-glucoside	Luteolin	Apigenin	Acacetin
Sample 1	0.32	1.22	0.046	0.23	0.48	0.0044	0.11	0.028
Sample 2	0.32	1.16	0.045	0.19	0.41	0.017	0.17	0.033
Sample 3	0.33	1.11	0.049	0.18	0.45	0.017	0.17	0.041
Sample 4	0.11	1.16	0.045	0.15	0.28	0.056	0.095	0.14
Sample 5	0.06	0.79	0.030	0.076	0.19	0.05	0.088	0.15
Sample 6	–	0.032	–	–	0.062	0.019	0.065	0.27
Sample 7	–	0.033	–	–	0.062	0.017	0.063	0.26
Sample 8	–	0.038	–	–	0.089	0.022	0.078	0.28
Sample 9	–	0.040	–	–	0.13	0.023	0.098	0.28
Sample 10	–	0.033	–	–	0.14	0.024	0.093	0.23
Sample 11	–	0.047	–	–	0.16	0.022	0.089	0.21

–, not detected

The concentrations of the three flavonoid glycosides in group 1 (samples 1, 2, 3) were slightly higher than in sample 4, while aglycone concentrations decreased slightly. There was no significant difference in the total quantity of flavonoids in group 1 compared with sample 4. The concentration of flavonoids in group 3 decreased, the corresponding concentration of aglycons increasing. The total quantity of the six flavonoids measured also reduced (Table 1). According to a preliminary experiment [14], we believe that the reason for the decrease in flavonoid glycosides in group 3 is that the heat-drying process in *Boju* activates the *β*-glucosidase present in *Boju*, acting on the flavonoid glycosides. The proportion of the various components in sample 4, simulating the traditional drying process of *Boju*, the hydrolysis of glycosides to aglycons and higher concentration of flavonoid glycosides is due to the lower temperatures when drying, inhibiting the activity of *β*-glucosidase.

### Purification of *β*-glucosidase from *Boju*

**Fig. 4:**
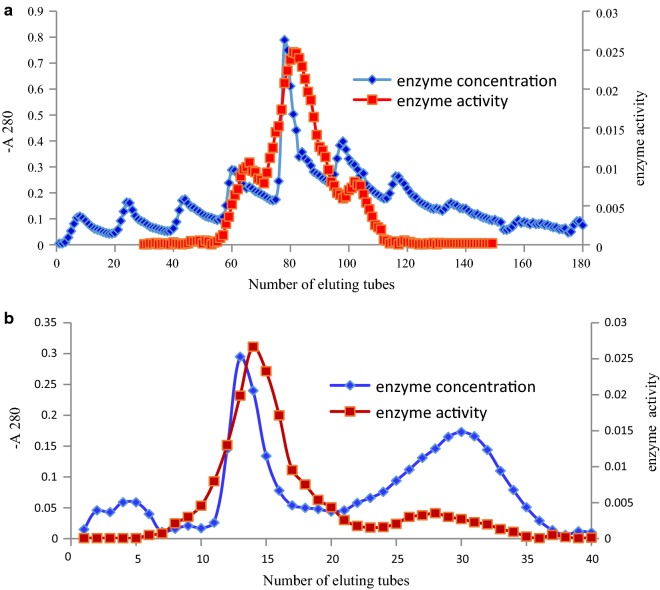
Purification of *β*-glucosidase obtained from *Boju*. **a** Chromatograms obtained by DEAE celluose-52 anion-exchange chromatography and **b** gel filtration from a Sephadex™ G-100 column

*β*-Glucosidase was purified from *Boju*. Approximately 91% of *β*-glucosidase activity was recovered from the precipitate within the 30–80% ammonium sulfate concentration fraction. Subsequently, the desalted concentrated active ammonium sulphate-fractions were loaded onto a DEAE celluose-52 anion-exchange chromatography column. The elution patterns from chromatography are shown in Fig. 4a. Salt buffer elution treatment resulted in better separation, with a minor protein peak that was eluted out with an elution buffer containing 1 M NaCl, reflecting *β*-glucosidase activity. The active fractions were pooled and dialyzed to remove NaCl, then concentrated by ultrafiltration for gel filtration. The elution pattern for gel filtration is shown in Fig. 4b. After Sephadex™ G-100 gel filtration, the *β*-glucosidase from *Boju* was purified to improve homogeneity. The purification steps and yields are displayed in Table 2. In the purification process, 0.087 units of the purified *β*-glucosidase were obtained. The recovery was 4.3%, with a specific activity of 0.124 U/mg and 24.8-fold purification efficiency.

**Table 2:** Summary of the procedures for purification of *β*-glucosidase from *Boju*

Step	Total protein (mg)	Total activity (U)	Specific activity (U/mg)	Purification (fold)	Activity recovery (%)
Crude extract	363	1.980	0.005	1	100
Ammonium sulfate precipitation	79	0.784	0.010	2.0	39.6
DEAE-cellulose-52 chromatography	8.1	0.402	0.040	9.9	20.3
Sephadex™G-100 gel filtration	0.7	0.087	0.124	24.8	4.3

### Enzyme purity determination and SDS-PAGE

**Fig. 5:**
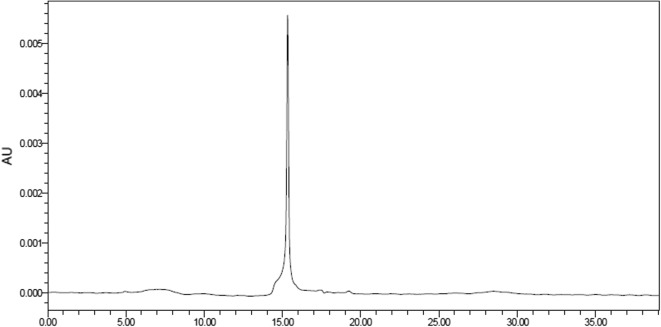
HPLC chromatogram activity peak from Sephadex™ G-100 gel filtration

**Fig. 6:**
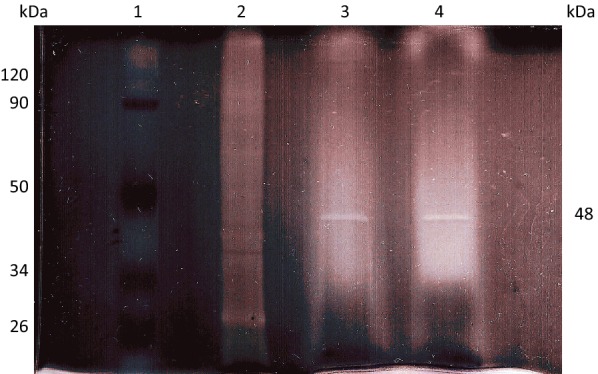
SDS-PAGE analysis (10% polyacrylamide) of protein samples from each purification step. Lane 1: protein markers; lane 2: 30–80% ammonium sulfate precipitation; lane 3: activity peak from DEAE celluose-52 chromatography; lane 4: activity peak from Sephadex™ G-100 gel filtration

HPLC and SDS-PAGE were used to evaluate purity of the isolated active enzyme. The isolated enzyme was obtained in a single peak by HPLC with good symmetry (Fig. 5). The SDS-PAGE profiles of the *β*-glucosidase-related fractions, including protein standards, the ammonium sulfate precipitate, and eluates from DEAE celluose-52 chromatography and Sephadex™ G-100 gel filtration are presented in Fig. 6. SDS-PAGE of the purified *β*-glucosidase under denaturing conditions revealed a single protein band of approximately 48 kDa (Fig. 6, lane 4). The *β*-glucosidase isolated from *Boju* is similar to the *β*-glucosidase subunit reported by Chiou et al. [18].

### Hydrolysis of substrates by isolated *β*-glucosidase

**Fig. 7:**
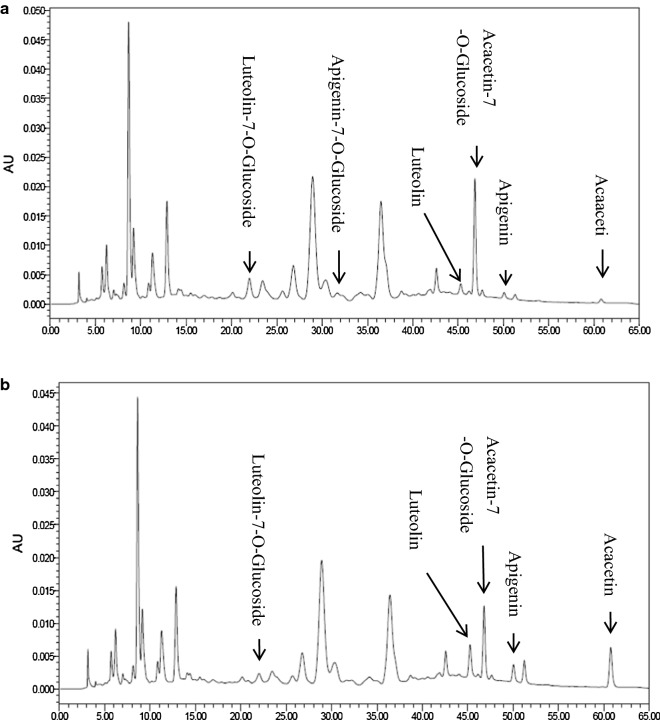
High-performance liquid chromatography of isoflavones in the substrate material after enzymatic reaction with the *β*-glucosidase from *Boju* at 50 °C, pH 5.0, for 60 min. **a** Incubation with the inactivated enzyme solution; **b** incubation with the native enzyme

We used the total flavonoids as a substrates for the isolated *β*-glucosidase. The luteolin-7-O-glucoside, apigenin-7-O-glucoside and acacetin-7-O-glucoside peaks in the hydrolytic product of the substrate were much reduced in *β*-glucosidase than in the experimental control (Fig. 7). However, after 65 min of hydrolysis, a considerable quantity of luteolin, apigenin and acacetin were formed. The results suggest that aglycones, namely, luteolin-7-O-glucoside, apigenin-7-O-glucoside and acacetin-7-O-glucoside were converted to luteolin, apigenin and acacetin by the enzyme. The results in Table 3 indicate that the quantity of luteolin, apigenin and acacetin increased with reduced concentrations of luteolin-7-O-glucoside, apigenin-7-O-glucoside and acacetin-7-O-glucoside because of hydrolysis by *β*-glucosidase.

**Table 3:** Concentrations of the principal flavonoids in the total flavonoid substrate and the hydrolytic product by hydrolysis of the *β*-glucosidase

Sample	μg/25.45 mg of sample					
	Luteolin-7-O-glucoside	Apigenin-7-O-glucoside	Acacetin-7-O-glucoside	Luteolin	Apigenin	Acacetin
Flavonoids substrate	3.019	0.322	8.050	0.859	0.317	0.202
Hydrolytic product	1.539	0	4.769	2.966	0.835	1.860

## Conclusions

*β*-Glucosidase was extracted and purified from *Boju*. This study confirmed that *β*-glucosidase can hydrolyze flavonoid glycosides to form the corresponding flavonoid aglycons. The results imply that *β*-glucosidase is the main factor causing hydrolysis of flavonoid glycosides during the processing of *Boju*. Due to the action of *β*-glucosidase, there were significant changes in the composition of different processing samples of *Boju*, suggesting that there are a number of differences in the efficacy and drug metabolism of *Boju* samples obtained by different processing methods. Therefore, future experiments need to study differences in the efficacy and drug metabolism of *Boju* samples using different processing methods.

In accordance with differences in origin and processing method, chrysanthemum morifolium is categorized into *ʻBojuʼ, ʻChujuʼ, ʻGongjuʼ, ʻHangju’* or *ʻHuaijuʼ.* This study confirmed that *β*-glucosidase derived from *Boju* can hydrolyze flavonoid glycosides to form the corresponding flavonoid aglycons. Therefore, we believe that *β*-glucosidase has a common role in the processing of medicinal chrysanthemums, suggesting that the relationship between *β*-glucosidase and change in composition during processing of *ʻChujuʼ*, *ʻGongjuʼ*, *ʻHangjuʼ* and *ʻHuaijuʼ* should also be studied.

## Data Availability

The datasets used and analysed during the current study are available from the corresponding author on reasonable request.

## Abbreviations

ρNPG: ρ-nitrophenyl-*β*-d-glucopyranoside
HPLC: high performance liquid chromatography
SDS-PAGE: sodium dodecyl sulfate-polyacrylamide gel electrophoresis
PCA: principal components analysis
PLS-DA: partial least squares regression with discriminant analysis
