# Correction to: Study of the mechanism of change in flavonoid composition in the processing of *Chrysanthemum morifolium* (Ramat.) Tzvel. ‘*Boju*’

**DOI:** 10.1186/s13065-020-00668-4

**Published:** 2020-03-05

**Authors:** Wei Zhang, Yafeng Zuo, Fengqing Xu, Tongsheng Wang, Jinsong Liu, Deling Wu

**Affiliations:** 1 School of Pharmacy, Anhui University of Chinese Medicine, No. 1 Qianjiang Road, Hefei, 230000 China; 2 School of Chinese Materia Medica, Bozhou University, No. 2266 Tangwang Road, Bozhou, 236800 China

## Correction to: BMC Chemistry (2019) 13:128 10.1186/s13065-019-0645-0

Following publication of the original article [1], the author reported an error in the reference citation. In Results and Discussion section, Zhang et al. [2] should be cited after the first and third sentences of the first paragraph under the sub-section “Determination of main components in different processed products of Boju”. The reference has been included in the list.
